# Development of an anti-HIV vaccine eliciting broadly neutralizing antibodies

**DOI:** 10.1186/s12981-017-0178-3

**Published:** 2017-09-12

**Authors:** Yousuf Ahmed, Meijuan Tian, Yong Gao

**Affiliations:** Department of Microbiology and Immunology, Schulich School of Medicine and Dentistry, The University of Western Ontario, London, ON Canada

**Keywords:** HIV-1, Diversity, Broadly neutralizing antibody, Polyvalent vaccine, B cell maturation

## Abstract

The extreme HIV diversity posts a great challenge on development of an effective anti-HIV vaccine. To solve this problem, it is crucial to discover an appropriate immunogens and strategies that are able to prevent the transmission of the diverse viruses that are circulating in the world. Even though there have been a number of broadly neutralizing anti-HIV antibodies (bNAbs) been discovered in recent years, induction of such antibodies to date has only been observed in HIV-1 infection. Here, in this mini review, we review the progress in development of HIV vaccine in eliciting broad immune response, especially production of bNAbs, discuss possible strategies, such as polyvalent sequential vaccination, that facilitates B cell maturation leading to bNAb response.

## Background

According to the WHO, there were ~36.7 million people worldwide living with HIV/AIDS by the end of 2015 and 2.1 million new HIV infections in 2015. In Canada, there were estimated 75,500 people living with HIV infection or AIDS at the end of 2014, a 9.7% increase from 2011, with more than 2500 people are newly infected each year. Unfortunately, even after over 30 years of intensive research, there is still no effective anti-HIV vaccine. This mini review will focus on the development of anti-HIV vaccine targeting elicitation of broadly neutralizing antibodies (bNAbs) against extremely diverse HIV strains.

## HIV diversity and vaccine development

The extreme genetic diversity of HIV, as a result of high baseline rates of viral mutation and replication, has been a great challenge for HIV vaccine development [1, 2]. There are two types of HIV: HIV-1, predominant throughout the world, and HIV-2, found primarily in West/Central Africa. HIV-1 contains four groups: M (main), O (outlier), N (non-M/non-O), and P (pending). Group M is further subdivided into 9 distinct subtypes [3] and numerous additional circulating recombinant forms (CRF) [4]. Viruses within the same subtype differ by up to 20%, within the highly variable *env* region by up to 38%. Furthermore, the virus continuously diversifies in infected individuals, resulting in the virus quasispecies varying up to 5% genetic difference in the same patient at different time points. These quasispecies compose of a unique and highly complex mixture of variants in infected individuals, and ultimately give rise to a highly diverse global virus population.

## Development of broadly effective anti-HIV vaccine

It is widely thought that an effective strategy to prevent HIV infection will likely come from T cell and B-cell mediated immunity, especially a broadly neutralizing antibody (bNAb) response against the Envelope (Env) protein. The power of bNAbs comes from their ability to recognize epitopes from a variety of viruses, i.e. tackling the extreme viral diversity, and their ability to protect in vivo at low plasma levels [2]. A safe vaccine eliciting bNAbs against HIV could be used to attenuate its spread.

To overcome HIV-1 diversity, one approach is to include different clades to develop a broadly protective polyvalent vaccine. However, early studies on polyvalent vaccine showed inconsistent results regarding elicitation of broad immune responses. Several early studies showed that a polyvalent vaccine, comprising a combination of multiple Env proteins, was better at eliciting broader immune responses than monovalent Env in both rabbits and macaques [5–7], while a clinical phase 2b trial of HVTN505 combining three envelope glycoproteins from clade A, B, and C *env* genes did not reduce either the rate of acquisition or set point viral load of new HIV-1 infections [7].

The HIV-1 T cell vaccine field has recently made some significant advances, such as a novel CMV vector developed by Louis Picker et al. [8], and a polyvalent HIV-1 mosaic antigen strategy which utilizes a genetic algorithm to design small sets of artificial intact viral proteins and collectively optimizes coverage of diverse potential epitopes in a targeted population for a given set size, or valency [9]. Vaccination of a Rhesus Macaque model showed that over 50% of SIVmac infections were effectively cleared in animals that were vaccinated with SIV antigens delivered by the CMV-vectored SIV vaccines [8]. Several recent studies have shown that polyvalent HIV-1 Mosaic antigens result in significantly greater breadth and potency of vaccine-elicited T-cell responses than do natural proteins in NHP studies [10–12].

## Broadly anti-HIV neutralizing antibodies

Anti-HIV bNAbs were discovered in the early 1990s when researchers found antibodies capable of neutralizing different virus subtypes [13]. Characterization of these responses has shown the bNAbs target sites include the conserved regions near the CD4 binding site (CD4bs) [13], the membrane-proximal external region (MPER) [14], and the base of the V3 and V1/V2 loops [15] of which some bNAbs are glycan-dependent [16–18]. Despite the early discovery of broadly neutralizing anti-HIV antibodies (bNAbs), including 447-52D (V3 loop), b12 (CD4 binding site), 17b (co-receptor binding site), 2G12 (viral glycan), 4E10 and 2F5 (gp41 MPER), enthusiasm for an Ab-based vaccine was limited based on the unusual characteristics of these bNAbs: 2G12 has three antigen combining sites, instead of the usual two [19]; 2F5 and 4E10 are self-reactive [20, 21]; and b12 is a phage-derived Ab generated by random pairing of heavy and light chains that may have never existed in nature [22]. However, recent development of single-cell antibody cloning techniques applied to plasma B cells of HIV infected patients uncovered variety of new bNAbs (Table 1), and detailed analyses of these antibodies indicated they are approximately 10- to 100-fold more potent and have an increased breadth compared with the original 4 isolates [23, 24]. To date, there have been a few clinical trials with anti-HIV bNAbs that are successful in reducing viral loads, most notably with 3BNC117 (a CD4bs-specific antibody) currently in phase 2 clinical trials [3–5]. Other studies also showed that passive infusion of NAbs could effectively protect macaques from vaginal SHIV challenge [25, 26]. These results suggest a role of Abs in HIV protection and control, but HIV has a tendency to accumulate mutations, making it a difficult target in vaccination strategies. Epitope mapping of the new, potent antibodies has invigorated the vaccine field by providing precise regions to target when designing new protein or subunit vaccine antigens to induce bNAbs [27]. However, even with this new wealth of information at hand, generating bNAbs with improved, redesigned antigens still prove to be problematic, and there are no appropriate immunogens/vaccination strategies that have been discovered to elicit an effectively protective Ab response.

**Table 1:** Characteristics of anti-HIV bNAbs

Env site	Antibody designation	Neutralization breadth, %	Neutralization potency, μg/ml	Length of CDR H3, a.a.	Somatic mutations %	Year of generation
CD4bs	b12^a^	35–75	2.82	18	17.3	1991
	HJ16	36	8.01	21	36.7	2010
	VRC01	88–93	0.09	14	38.8	2010
	VRC02	90–91	0.13	14	34.9	2010
	VRC03	51–59	0.08	16	34.9	2010
	PGV04	77–88	0.14	16	38.2	2011
	CH31	84–91	0.02	15	31.9	2011
	CH33	90	0.24	15	31.9	2011
	NIH45-46	84–86	0.08	18	44	2011
	3BNC117	86–92	0.06	12	36.9	2011
	12A12	92–96	0.07	15	34	2011
	VRC23	65–80	0.58			2013
V1/V2 loop	PG9	77–83	0.08	30	15.4	2009
	PG16	73–79	0.02	30	16.8	2009
	PG145	78	0.29	33	22.8	2011
	CH01	46	3.75	24	23.3	2011
V1/V2 loop	2G12^a^	28–39	1.45	16	33.6	1994
	PGT121	70	0.03	26	21.2	2011
	PGT128	72	0.02	21	27.9	2011
CD4i/V3	3BC176	64	12.8	19	29.4	2012
gp41 MPER	2F5^a^	55–67	1.44	24	15.2	1992
	4E10^a^	85–100	1.62	20	15.6	1994
	Z13	35	40	19	21	2001
	10E8	98–99	0.25	22	22.1	2012
gp120/gp41	PGT151-155	64–66	0.008–0.012	28		2014
Interface	Interface	67	0.87	9		2011

^a^First generation of bNAb

## Development of immunogens and vaccination strategies to elicit anti-HIV bNAbs

It has been reported that, during chronic infection, potent and cross-reactive bNAbs that are capable of neutralizing heterologous viruses of diverse subtypes develop in a small portion of HIV-1 infected individuals [28–31]. The effective humoral responses are slow, with NAbs to the initial viral strain appearing after ~12 weeks, and broad NAbs (in 10–30% of individuals) after 2–4 years [30, 32–34]. The development of bNAbs was shown to correlate with high plasma viremia and could result from evolving antigen exposure over many years that has allowed sufficient somatic hypermutation in the B-cell receptors (BCRs) and focuses the B-cell response to the conserved neutralization sites on Env [30]. Therefore, delayed bNAb response might be attributed to the slow, antigen-dependent affinity maturation process. Abs typically accumulate mutations in the complementarity determining region (CDR) loops, i.e. the typical antigen contact region [35]. Whereas most human Abs that have undergone affinity maturation carry 15–20 somatic mutations, potent anti-HIV bNAbs carry 40–110 mutations. Reversion of these mutations to the germline sequence drastically reduces their neutralizing potency and breadth [36–40]. These findings suggest that bNAb-producing B cells are the products of clonal evolution which coined the term “B cell maturation”. Thus, selecting the right combination of immunogens and vaccination strategy is crucial for induction of these bNAb-producing B cells via continual increase in affinity-driven selection in the germinal centers (GCs).

A vaccine strategy that aims to mimic the diverse antigenic exposure experienced during natural infection may generate NAbs of greater breadth and potency. Morner et al. achieved greater focusing of the immune response on conserved regions of Env by sequential vaccination of macaques with gp120 core protein followed by trimer boosting [41]. Similarly, the administration of sequential rather than single or mixed patient-derived gp140 Env genes in rabbits showed a marginal improvement in breadth of neutralization [42]. Sellhorn et al. recently described a novel assembly of gp140 heterotrimers which had gp140 subunits from two different genetic sources and improved potency of NAb responses in rabbits as compared to homotrimeric equivalents [43]. Wang et al. also showed through vaccinating mice with three gp120 variants that sequential vaccination is preferred over a cocktail for induction of cross-reactive Abs focused on the shared CD4 binding site epitope [44].

**Fig. 1:**
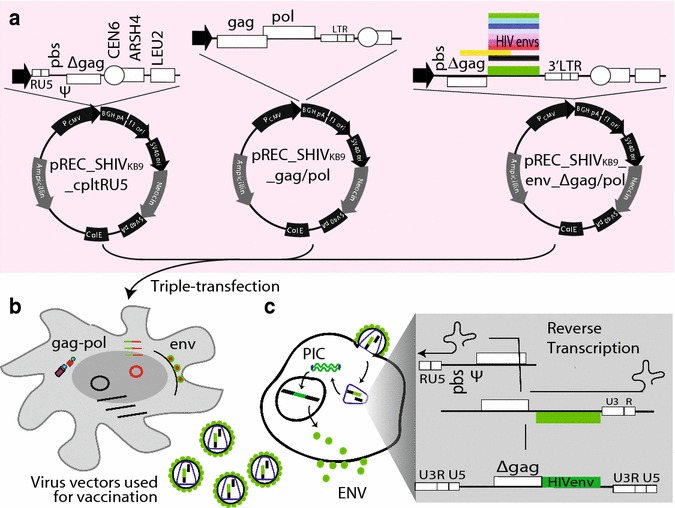
SHIVenv vaccine system. **a** The vaccine vector is derived from 293T transfections with three DNA plasmids: *1* pREC_SHIV_KB9_ _gag/pol vector containing gag and pol coding sequence from SHIV_KB9_ but lacking the LTRs or RNA packaging elements; *2* pREC_SHIV_KB9__env_Δgagpol vector which contains the RNA packaging signal, *env* and 3′LTR, but lacks 5′LTR, gag and pol sequences; *3* pCMV_SHIV_KB9__ cpltRU5 which contains only the 5′LTR, primer binding site, and the RNA packaging signal. **b** Generation of SHIVenv virus-like particles as HIV vaccine candidate through triple transfection. **c** Production of defective SHIV-1 proviral DNA and Env glycoproteins by pseudotyped virus containing the two complementary subgenomic viral RNAs

In order to develop an effective and safe polyvalent anti-HIV vaccine, our group has established a SHIV (and HIV) vaccine system which is composed of three DNA plasmids containing complementary subgenomic viral DNAs (Fig. 1), to investigate the optimal polyvalent vaccination strategy. Co-transfection of these three plasmids can produce SHIV (or HIV) virus-like particles (VLPs) containing two distinct subgenomic RNAs that are complementary to each other producing proviral DNA when infecting susceptible cells. The integrated proviral DNA is devoid of most viral coding sequences preventing any viral propagation but retains the *env* gene for continuous gp120/gp41 expression at the cell surface. The resultant VLPs are morphologically indistinguishable from a wild type virus, and can elicit direct responses by binding surface Ig on B cells, as well as bind target cells through CD4 and CCR5, induce all of the conformational changes, and expose all hidden epitopes. After entering the target cells, expression of HIV-1 Env glycoproteins should also promote proteosomal processing and Env peptide presentation through MHC class I, thus these VLPs can induce both humoral and cellular immune responses. Production of these polyvalent Env-based vaccines may initially appear intuitive, e.g. cloning of multiple HIV-1 *env* sequences, but until recently this type of cloning represented one of the greatest obstacles to this endeavor. Simply put, unique and conserved restriction sites do not exist for cloning the extremely diverse HIV-1 *env* gene. We have developed a yeast-based cloning system to clone any HIV-1 *env* into a common HIV-1 or SHIV backbone through sequence homology recombination/gap repair [45–47]. This system was designed for rapid yeast-based cloning and we have introduced >130 HIV-1 *env* genes with balanced representation from different HIV-1 subtypes (e.g. A, B, C, D) for our polyvalent vaccine construction. Furthermore, the continuous Env protein expression (but not the viral particles) was successfully detected in an in vitro susceptible cell culture.

We have tested our polyvalent anti-HIV vaccine in a human CD4 B cell transgenic mouse model which was established to express human CD4 receptor on the surface of B cells, thus can mediate HIV binding, and expose the hidden epitopes on viral gp120. We have tested 25 primary isolates (subtype A, B, C, and D) derived functional Envs and 25 nonfunctional inter-subtype recombinant Env-based VLP vaccines in the human CD4 B cell transgenic mice. The results showed that the sequential vaccination with the 25 functional primary Env-based polyvalent vaccine elicited broader NAb response than any other vaccine combination and vaccination strategy when examining inhibition of a number tier 1 and 2 viruses with different HIV-1 subtype Envs (e.g. A, B, C, and D). More importantly, with each increase in the diversity and number of vaccines (i.e. sequential vaccination), we observed a greater breadth in humoral responses (unpublished data). These results suggest that continuous stimulation with diverse HIV-1 Env immunogens will modulate B cell affinity maturation, educate the immune response to focus on certain regions (most likely on the conserved regions) in Env, and finally have the best success of generating broad NAb response.

## Conclusions

A major obstacle for HIV vaccine development is the extreme virus diversity. Although a number of bNAbs have been isolated from HIV patients, the vaccine and procedures capable to elicit such a response remain a mystery. The sequential vaccination with multiple immunogen variants might favor B cell re-circulation within GCs for additional rounds of affinity maturation. This may promote the B cell response to focus on the most conserved regions of immunogens through positive selection, resulting in more potent and broader antibody responses.
